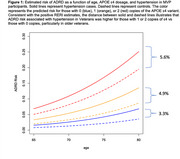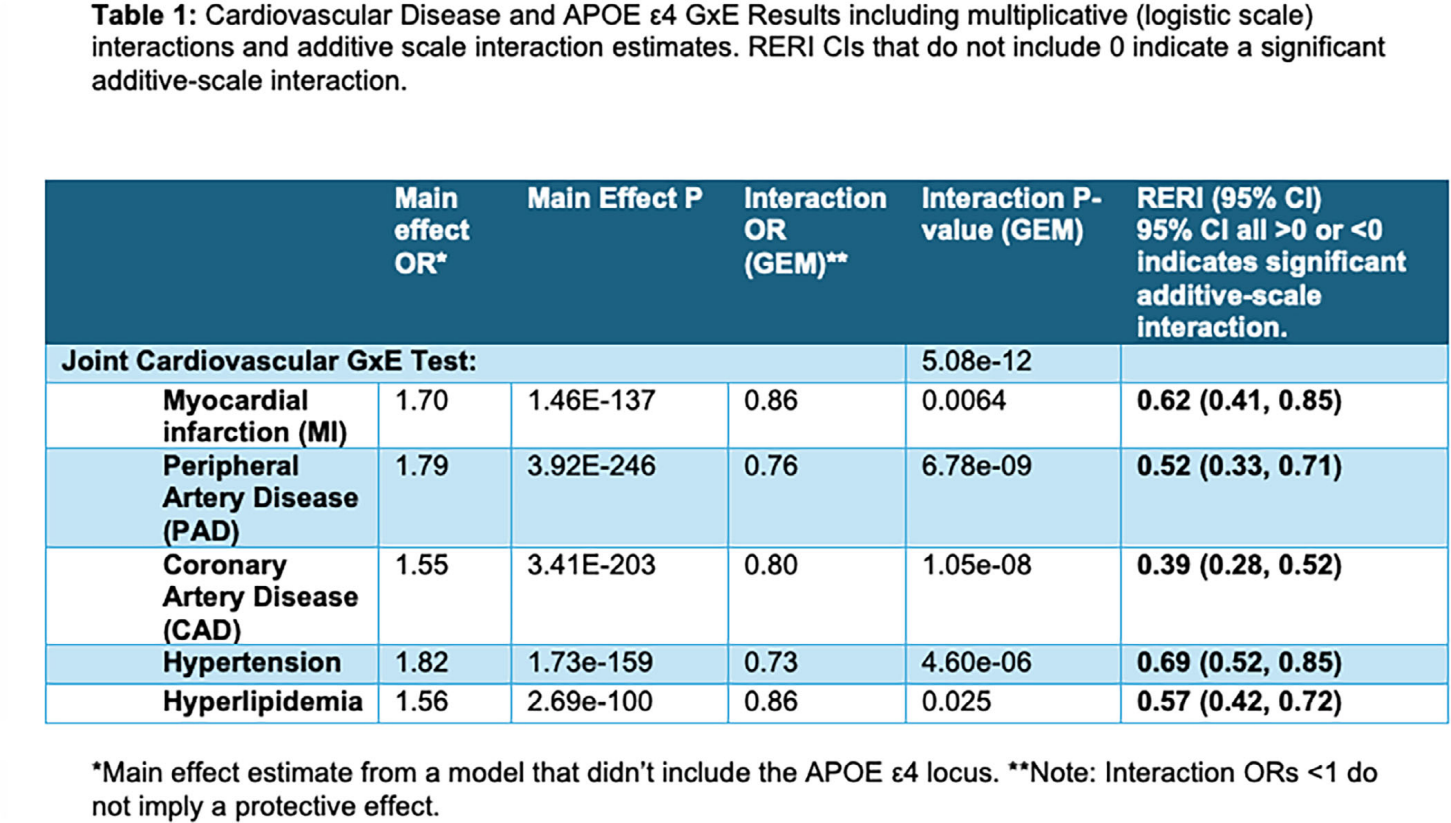# Alzheimer's Disease and Related Dementias among Aging Veterans: Examining Gene‐by‐Environment Interactions with Cardiovascular Diseases

**DOI:** 10.1002/alz70855_105948

**Published:** 2025-12-24

**Authors:** Mark W. Logue, Rui Zhang, Mark Miller, Richard Sherva, Brendan M. Despard, Kelly Harrington, Jennifer Fonda, Victoria C. Merritt, Matthew S. Panizzon, Richard L. Hauger, Erika Wolf, J. Michael Gaziano, Million Veteran Program

**Affiliations:** ^1^ Department of Psychiatry, Boston University Chobanian & Avedisian School of Medicine, Boston, MA, USA; ^2^ National Center for PTSD, VA Boston Healthcare System, Boston, MA, USA; ^3^ Biomedical Genetics, Department of Medicine, Boston University Medical School, Boston, MA, USA; ^4^ VA Center for Data and Computational Science (C‐DACS), Boston, MA, USA; ^5^ VA Boston Healthcare System, Boston, MA, USA; ^6^ Center of Excellence for Stress and Mental Health, VA San Diego Healthcare System, San Diego, CA, USA; ^7^ Department of Psychiatry, University of California San Diego, La Jolla, CA, USA; ^8^ Center for Behavior Genetics of Aging, University of California, San Diego, La Jolla, CA, USA; ^9^ University of California San Diego, La Jolla, CA, USA; ^10^ Center for Excellence for Stress and Mental Health (CESAMH), VA San Diego Healthcare System, San Diego, CA, USA; ^11^ Division of Aging, Brigham & Women's Hospital, Harvard Medical School, Boston, MA, USA; ^12^ Million Veteran Program (MVP) Coordinating Center, VA Boston Healthcare System, Boston, MA, USA

## Abstract

**Background:**

Cardiovascular diseases (CVDs) such as peripheral artery disease (PAD) and coronary artery disease (CAD) are risk factors for Alzheimer's disease (AD) and related dementias (ADRD). The *APOE*‐ε4 variant, which codes for a cholesterol transporter protein, is the largest AD genetic risk factor, increases LDL cholesterol and triglycerides, and augments the risk of cardiovascular disease. In this study of participants in the US Department of Veterans Affairs’ Million Veteran Program (MVP), we examined the interactive effects of *APOE*‐ε4 status with CVDs (PAD, CAD, myocardial infarction, hypertension, and hyperlipidemia) on ADRD prevalence.

**Method:**

Our cohort included MVP participants of European ancestry age 65 and older with available genotype data (*n* = 11,112 ADRD cases and 170,361 controls). Cross‐sectional logistic regression analyses were performed using the GEM (Gene–Environment interaction analysis in Millions of samples) software package and included fitting an omnibus test for gene by environment (GxE) interactions between additively‐coded ε4 and the CVDs as a group, followed by GxE analysis of individual CVDs. Additive‐scale interactions were measured using the *Relative Excess Risk due to Interaction* (RERI) statistic. ADRD was derived from International Classification of Diseases (ICD) codes using our validated algorithm. We used validated algorithms for MI and PAD identified in the VA's Centralized Interactive Phenomics Resource (CIPHER). CAD, hypertension, and hyperlipidemia cases were identified using Phecodes.

**Result:**

CVDs showed both strong main‐effect associations with ADRD (ORs 1.55 to 1.82, all *p* < 10^99^; see Table). Both the omnibus test (*p* = 5x10^‐12^) and the individual CVD interaction terms were significant (*p* from 7x10^‐8^ to 0.025). RERI estimates indicated significant positive additive‐scale interactions (see example figure illustrating additive hypertension x ε4 interaction).

**Conclusion:**

These additive‐scale interactions are more directly interpretable than multiplicative‐scale interactions. They indicate that the prevalence of ADRD associated with cardiovascular disease increases with the number of inherited *APOE*‐ε4 alleles (e.g. from 3.3% greater ADRD frequency associated with hypertension at age 80 for those with 0 ε4 copies to 5.6% for those with 2 copies; see Figure). Combining genetic testing with information about health comorbidities could contribute to more accurate dementia risk assessment within the Veteran population, and likely within other populations as well.